# Epidemiological dynamics of Leishmania (Sauroleishmania) tarentolae and Trypanosoma platydactyli in reptile hosts and sand flies: from isolation to genome assembly

**DOI:** 10.1099/mgen.0.001567

**Published:** 2025-12-09

**Authors:** Livia Perles, Jairo Alfonso Mendoza-Roldan, Marcos Antonio Bezerra-Santos, Petr Volf, Letterio Giuffrè, Domenico Giosa, Sara Epis, Claudio Bandi, Orazio Romeo, Domenico Otranto

**Affiliations:** 1 Department of Veterinary Medicine, Università degli Studi di Bari Aldo Moro, 70124 Bari, Italy; 2 Department of Parasitology, Faculty of Science, Charles University, 12800 Prague, Czechia; 3 Department of Chemical, Biological, Pharmaceutical and Environmental Sciences, University of Messina, 98166 Messina, Italy; 4 Department of Biosciences, Pediatric CRC “Romeo ed Enrica Invernizzi”–University of Milan, 20133 Milan, Italy; 5 Department of Veterinary Clinical Sciences, City University of Hong Kong, 518057 Hong Kong, PR China

**Keywords:** epidemiology, phlebotomine sand flies, reptile trypanosomatids, whole-genome sequencing

## Abstract

*Leishmania* (*Sauroleishmania*) *tarentolae* and *Trypanosoma platydactyli* are reptile-associated trypanosomatids transmitted by *Sergentomyia minuta* sand flies, posing challenges for accurate diagnosis due to the fact that they often occur in sympatry. This study aimed to isolate and characterize new strains of these parasites from reptiles and sand flies using morphological, molecular and genomic approaches. Fifty-five reptiles were captured in Apulia, Italy, and sand flies were collected and dissected under a surveillance framework. Blood samples and gut contents were cultured in Schneider’s Drosophila (SC) and Tobie-Evans (TEv) media. Two positive cultures underwent whole-genome sequencing, and a new conventional PCR (cPCR) protocol targeting the *β-tubulin* gene was developed. *T. platydactyli* was isolated from 27% of *Tarentola mauritanica* geckos using SC medium and 12.5% with TE, while *L*. (*S*.) *tarentolae* was isolated from 4.15% of geckos exclusively with SC. Cytology confirmed *T. platydactyli* in 25% of gecko blood smears. cPCR revealed *T. platydactyli* in 18.75%, *L*. (*S*.) *tarentolae* in 12.5% and co-infections in 14.6%. No infections were found in *Podarcis siculus* or *Hemidactylus turcicus*. Out of 208 *S*. *minuta* sand flies tested, 19 (9.1%) were positive for *T. platydactyli*, 30 (14.4%) for *L*. (*S*.) *tarentolae*, and 15 (7.2%) were co-infected with both. The newly developed cPCR assay robustly differentiated these parasites in both reptile and sand fly samples. Monitoring of natural infections in geckos revealed persistent, low-level *L*. (*S*.) *tarentolae* infections, detectable only by molecular methods, in contrast to the intermittent parasitaemia of *T. platydactyli*, which was detectable by cytology and culture. Kimura 2-parameter (K2P) divergence profiles indicate no evidence of a recent mobilome in *T. platydactyli*, whereas *L*. (*S*.) *tarentolae* retains a small but detectable fraction of low-divergence transposable-element copies (≤5–10% K2P; <0.05% of the genome). These findings confirm the sympatric circulation of *L*. (*S*.) *tarentolae* and *T. platydactyli* in geckos and sand flies in southern Italy, highlighting *T. mauritanica* as the most common reptilian host. The successful isolation and genome assembly of these trypanosomatids, along with the newly developed molecular tool, lay a solid foundation for future epidemiological and comparative genomic investigations, emphasizing the role of reptilian hosts in maintaining trypanosomatid diversity.

**Impact Statement**

Trypanosomatids such as *Leishmania* and *Trypanosoma* species are parasitic protozoa with diverse transmission strategies and complex ecological dynamics. In this study, we provide an integrated genomic and ecological investigation of *Leishmania* (*Sauroleishmania*) *tarentolae* and *Trypanosoma platydactyli*, two reptile-associated trypanosomatids found in lizards and sand flies in southern Italy. We describe their co-infection patterns, isolate new strains, develop a novel conventional PCR assay to detect single and mixed infections and analyse their genomes, providing a valuable resource for future studies on trypanosomatid biology and host–parasite interactions. Our results highlight differences in parasitaemia, infectivity and culture profiles between the two species. We also show that using Kimura 2-parameter (K2P) divergence profiles, no recent mobilome was detected in *T. platydactyli* (K2P), while *L*. (*S*.) *tarentolae* showed a small set of low-divergence elements. Our results contribute to the understanding of trypanosomatid biology by combining field parasitology, diagnostic innovation and comparative genomics.

## Data Summary

The authors confirm that all supporting data, code and protocols have been provided within the article or through supplementary data files.

## Introduction

Protozoa from the Trypanosomatidae family are among the most evolutionarily successful parasites, adapting to various hosts, including invertebrates and vertebrates [1]. Research on trypanosomatids has primarily focused on mammal-associated species, such as *Trypanosoma cruzi* and *Leishmania infantum*, which pose a significant health burden in tropical, subtropical and temperate regions [2,3]. In contrast, *Leishmania* (*Sauroleishmania*) *tarentolae*, a reptile trypanosomatid considered non-pathogenic to mammals, has been relatively understudied and only recently has it attracted the attention of researchers for its biotechnological applications [4]. Following the detection of *L*. (*S*.) *tarentolae* in a mummy [5], as well as in the blood of humans [6,7] and dogs [7,9], the potential interactions with the pathogenic *L. infantum* have been further investigated [9,10]. In experimentally infected dogs, *L*. (*S*.) *tarentolae* was able to infect and persist, being also immunogenic and inducing antibody production [11].

Despite the growing number of studies on *L*. (*S*.) *tarentolae*, many aspects of this trypanosomatid remain unknown, including its biological life cycle, pathogenicity and overall biology. In addition, there are no standardized protocols for isolating or maintaining *L*. (*S*.) *tarentolae* cultures. For example, the species was previously isolated from *Sergentomyia minuta* gut content and gecko blood using Tobie-Evans (TEv) medium [8,9], and cultures were maintained using RPMI-1640 medium or Schneider’s Drosophila (SC) medium, with or without FBS [12]. Due to the difficulties in obtaining new isolates, only three genomes of *L*. (*S*). *tarentolae* are currently available in GenBank (available from https://www.ncbi.nlm.nih.gov/datasets/genome/?taxon=5689https://www.ncbi.nlm.nih.gov/datasets/genome/?taxon=5689; accessed on 28 September 2025), exhibiting a significant size discrepancy (~5 million base pairs), likely due to missing repeat sequences in earlier assemblies [13,14]. Therefore, further studies are needed to fully understand the complexity and variability of the *L*. (*S*.) *tarentolae* genome.

In areas where *L*. (*S*.) *tarentolae* circulates among reptiles, *Trypanosoma* spp. has also been detected [9]. For instance, *Trypanosoma platydactyli* (Catouillard 1909) was described from *Tarentola mauritanica* geckos, complicating investigations of *Leishmania* spp. in Mediterranean reptiles, as the cultured flagellated forms are morphologically similar [15]. Based on this, *T. platydactyli* was previously synonymized with *L*. (*S*.) *tarentolae*, until molecular and morphological studies confirmed the validity of both species [16]. Nonetheless, co-infections by both parasites were diagnosed in geckos in Malta by blood culturing and xenodiagnoses in *S. minuta* [17], and in Apulia, Italy, with *L*. (*S*.) *tarentolae* detected molecularly and *Trypanosoma* sp. detected by cytology [8,9]. Similar to *L*. (*S*.) *tarentolae*, many aspects of *T. platydactyli* life cycle, pathogenicity and biology remain poorly understood, with a single genome assembly available (available from https://www.ncbi.nlm.nih.gov/datasets/genome/?taxon=3041299; accessed on 28 September 2025) [1]. The genome of *T. platydactyli* has been used to explore evolutionary relationships within Trypanosomatidae, by comparing it with those of pathogenic and non-pathogenic species [1].

Since morphological identification of reptile trypanosomatids is challenging, especially in cases of co-infections, molecular diagnostic tools are essential for distinguishing between species. Moreover, there is a pressing need for more information on culture media suitable for the isolation and maintenance of these parasites, as well as for generating new genome assemblies to better understand their biology and diversity. Therefore, this study aimed to: (i) evaluate two different culture media for the isolation and maintenance of both agents from reptile blood and sand fly gut contents; (ii) generate new genome assemblies for both species through reference-guided variant calling, consensus correction and repeat landscape characterization using a lineage-specific Trypanosomatidae library; (iii) develop and validate a new conventional PCR (cPCR) protocol for detecting single and co-infections by *L*. (*S*.) *tarentolae* and/or *Trypanosoma* sp. in reptile blood and sand flies and (iv) monitor natural infections by *L*. (*S*.) *tarentolae* and *T. platydactyli* in lizards over time.

## Methods

### Ethical approval

Protocols for the collection of reptiles were approved by the ethical committee of the Department of Veterinary Medicine of the University of Bari, Italy (Prot. Uniba 12/20), and authorized by the Ministry for Environment, Land and Sea Protection of Italy (Approval No. 0073267/2019), the Societas Herpetologica Italica and the Istituto Superiore per la Protezione e la Ricerca Ambientale (Approval No. 71216).

### Reptile capturing and processing

Fifty-five reptile specimens (*n*=48 *T*. *mauritanica*, *n*=6 *Podarcis siculus* and *n*=1 *Hemidactylus turcicus*) were collected over 7 months (May–November 2024) in a dog shelter in Apulia, Italy (41°03′04.3″N, 16°53′39.7″E), where *L*. (*S*.) *tarentolae* was previously detected by Real Time Quantitative PCR (qPCR) and *Trypanosoma* sp. by cytology in geckos [8]. Animals were captured by lassoing or by hand, and data were recorded in specific files. A small amount of blood (∼100 µl) was obtained by cardiocentesis from subadult and adult animals. Samples (~5 µl) were used for smears (stained with Diff-Quik), cultivation (~50 µl), and the remaining blood was stored at −20 °C until molecularly processed.

### Sand flies

*S. minuta* were collected under a framework of a surveillance study [18], using CDC (Centers for Disease Control and Prevention) light traps at the same dog shelter in Apulia, Italy. Briefly, alive females were dissected with a drop of saline solution, and the gut was observed under a microscope to determine the presence of flagellates, according to procedures previously described [19]. The gut content of positive females (i.e. with promastigotes or trypomastigotes present) was cultivated, and negative gut contents were stored in 70% ethanol for molecular analyses.

### Culture

For trypanosomatid isolation, gecko blood samples and the gut content of positive sand fly females were incubated in blood agar with 1 ml of TEv medium (supplemented with fluorocytosine 250 µg ml^−1^, gentamicin 250 µg ml^−1^ and FBS 5%), according to procedures previously described [20], or (SC) medium supplemented with streptomycin–penicillin 650 µg ml^−1^, FBS 10% and male urine 10%, without oxygen [21]. Cultures were maintained at 26 °C, evaluated every week and were considered negative after 35 days of inoculation. Positive cultures were subcultured (i.e. until passage 2) and frozen at −80 °C for genomic analyses.

### Genomic DNA extraction, library preparation and whole-genome sequencing

Genomic DNA (gDNA) from one isolate of *L*. (*S*.) *tarentolae* and one of *T. platydactyli* at passage 2 was extracted using the DNeasy Blood and Tissue Kit (QIAGEN, Germany), according to the manufacturer’s instructions. DNA quality and quantity (260/280 and 260/230 nm ratios) were assessed with a NanoDrop™ 2000c microvolume UV–Vis spectrophotometer (Thermo Fisher Scientific, USA) and an Agilent Fragment Analyzer System (Agilent Technologies, USA). Whole-genome, 150 bp paired-end libraries were prepared with the NEBNext^®^ Ultra™ II DNA Library Prep Kit for Illumina (New England Biolabs, USA), including fragmentation, end-polishing, A-tailing, adapter ligation and size selection. Purification steps used the AMPure XP system (Beckman Coulter Life Sciences, USA). Libraries were quality-checked on the Agilent system, quantified by Qubit, pooled and sequenced on an Illumina NovaSeq Plus platform at Novogene UK.

### Genome assembly, validation and annotation

FASTQ results were analysed to remove low-quality bases and adapter contaminations using fastp [22], and results were visualized with FastQC v0.12.1. Specifically, fastp v0.23.2 was run with automatic adapter detection and removal (--detect_adapter_for_pe), duplicate removal (--dedup), quality filtering (minimum Phred score 20; -q 20) and a minimum post-trim read length of 50 bp (-l 50); default parameters were used otherwise.

Canonical k-mers (k=32) were counted with Jellyfish v2.2.10 (https://github.com/gmarcais/Jellyfish). To reduce the effect of high-copy kinetoplast DNA, k-mers with multiplicity >1,000 were excluded from histograms prior to modelling. K-mer spectra were analysed with GenomeScope 2.0 (https://github.com/tbenavi1/genomescope2.0). For *L*. (*S*.) *tarentolae*, haploid (*P*=1) and diploid (*P*=2) models were tested; the haploid model provided the best fit to the data, consistent with the known reduced heterozygosity, clonality and widespread loss of heterozygosity/aneuploidy in these parasites [23,24]. Similarly, for *T. platydactyli*, both models were also tested, as species with unimodal spectra consistent with haploidy (e.g. *Trypanosoma theileri*, *Trypanosoma melophagium*) [25] coexist with species exhibiting strong heterozygous peaks consistent with diploidy (e.g. *T. cruzi*) [26,27]. The ploidy parameter was ultimately chosen based on histogram shape, and a haploid model (*P*=1) was applied to unimodal distributions. Model outputs included haploid genome size, average k-mer coverage, sequencing error rate, duplication rate and the proportion of unique versus repetitive sequence.

Processed reads were assembled by reference to the genome of *L*. (*S*.) *tarentolae* (GCA_033953505.1) and *T. platydactyli* (GCA_030849675.1) by Burrows–Wheeler Aligner (BWA-MEM) v0.7.18-r1243 [28]. Post-alignment processing was performed with SAMtools v1.21 [29], including sorting and indexing of BAM (Binary Alignment Map) files and coverage computation (i.e. samtools sort, samtools index and samtools depth). To minimize biases from regions with low sequencing depth, a coverage threshold of 10× was applied based on samtools depth. Variant calling was performed with BCFtools v1.19 [30] (bcftools call -m --ploidy 1), as supported by k-mer spectrum analysis. The resulting VCF (Variant Call Format) files were normalized against the reference sequence using bcftools norm -m -both and sorted with bcftools sort. Variant statistics were obtained with bcftools stats. Per-site read depth was computed from INFO/DP4 as the sum of forward and reverse counts for reference and alternate alleles. The median site depth was 210 for *T. platydactyli* and 73 for *L*. (*S*.) *tarentolae*. Depth thresholds were defined as 0.3×–3.0×the median (63–630 and 22–219, respectively).

To assess variant call reliability for both species, 30 randomly selected SNPs and 15 INDELs (insertion-deletion) were manually inspected in Integrative Genomics Viewer v2.19.1 (IGV). All inspected SNPs showed balanced, high-quality support on both strands, corroborating the initial filtering thresholds. For *T. platydactyli,* some INDELs showed borderline support in repetitive regions, which are inherently more error-prone. Therefore, an additional round of stringent filtering was applied to retain only high-confidence variants. SNPs were required to have QUAL (Phred-scaled quality score) ≥30, MQ (Mapping Quality) ≥40, MQ0F (Mapping Quality Zero Fraction) ≤0.10, VDB (Variant Distance Bias) >1×10⁻⁶ and ≥3 alternate reads on each strand (DP4). INDELs were required to have QUAL ≥50, mapping quality ≥40, MQ0F ≤0.10, VDB >1×10⁻⁶, ≥4 alternate reads on each strand (DP4 - Read Depth per Allele per Strand) and IDV (IDentifier Variant) ≥5 and IMF (Internal Minor Allele Frequency) ≥0.5. This two-step approach (i.e. initial liberal calling followed by manual inspection and strict re-filtering) ensured a conservative and high-quality final call set for downstream analyses. The final filtered variants were applied to the reference genome using bcftools consensus v1.19 (default parameters), generating corrected assemblies. No masking options were applied, and overlapping sites were automatically resolved by bcftools.

Assembly statistics (e.g. N50, G+C content and total size) were computed using QUAST v5.3.0 [31]. Genome completeness of both trypanosomatids was evaluated using BUSCO v5.8.2 (Benchmarking Universal Single-Copy Orthologs) [32] in genome mode, with lineage-specific datasets from OrthoDB v12. For *T. platydactyli*, the trypanosoma_odb12 dataset was used, which contains 5,397 single-copy orthologs specific to the *Trypanosoma* genus. For *L*. (*S*.) *tarentolae*, the leishmaniinae_odb12 dataset was used, comprising 6,640 genes representative of the *Leishmania* genus. BUSCO was run with default parameters, and completeness was assessed by summarizing the proportions of complete (single-copy or duplicated), fragmented and missing BUSCOs. Repetitive and transposable elements (TEs) were identified and soft-masked using RepeatMasker v4.1.6 with the lineage-specific library TEs_trypanosomatids [33], which includes the newest curated TE families characterized in trypanosomatids. Masking was performed using the options -engine rmblast, -lib, -xsmall and -nolow to generate a soft-masked genome for downstream analyses while excluding simple and low-complexity repeats. Unclassified or unannotated repeats were not retained or reported as a separate ‘Unknown/Unclassified’ category. The relative ages of TE copies were inferred from Kimura 2-parameter (K2P) divergence values computed with the calDivergenceFromAlign.pl script from the RepeatMasker package. Divergence distributions were summarized using a Python workflow that computes the genomic proportion of masked bases per TE group across bins of 0–2%, 2–5%, 5–10%, 10–20% and >20% from the consensus sequences. Comparative repeat landscapes were used to visualize divergence profiles and assess potential differences in the timing of TE activity between *L*. (*S*.) *tarentolae* and *T. platydactyli*.

### Molecular biology and new PCR protocol design

gDNA was extracted from reptile blood samples using the QIAamp DNA Micro Kit (QIAGEN, Hilden, Germany), from the thorax and abdomen of female sand flies using an in-house method, as previously described [34], and from cultures using the DNeasy Blood and Tissue Kit (QIAGEN, Hilden, Germany), according to the manufacturer’s instructions. gDNA obtained from all different samples was submitted to PCR analyses.

Whole-genome sequencing results were used for the development of a cPCR protocol to differentiate both species in blood samples and sand flies. A selected region of *β-tubulin* from both genomes was aligned for cPCR primer design using the PrimerQuest™ tool (available at: https://www.idtdna.com/pages/tools/primerquest). The primer’s specificity was analysed *in silico* using the basic local alignment search tool (blast; http://blast.ncbi.nlm.nih.gov/Blast.cgi). To assess the limit of detection (LOD) of the designed primers, serial dilutions of template DNA were prepared. A total of eight 10-fold serial dilutions were performed from gDNA extracted from culture (i.e. *T. platydactyli* and *Leishmania tarentolae*), ranging from 10 ng µl^−1^ to 1 fg μl^−1^. Each dilution was tested in triplicate to ensure reproducibility of the results. All cPCR primers, reaction mix and thermocycling conditions are shown in Table S1 (available in the online Supplementary Material). PCR products were visualized by electrophoresis on a 2% agarose gel. The LOD was determined as the lowest DNA concentration that yielded a visible band on the gel under the specified conditions. Negative controls [i.e. no-template control (NTC) and gDNA extracted from a negative *T. mauritanica* and one *S. minuta* from the laboratory colony] were included in each PCR reaction to rule out contamination and unspecific amplification. For assessing assay specificity, amplicons of the expected size obtained from biological samples were purified and sequenced in both directions using the Big Dye Terminator v.3.1 chemistry in a 3130 Genetic Analyzer (Applied Biosystems, Foster City, CA, USA) on an automated sequencer (ABI-PRISM 377). All lizard blood samples (i.e. 55 reptiles) and *S. minuta* sand flies (i.e. 66 engorged females and 142 non-engorged females) were submitted to the new cPCR under the conditions described in Table S1 to evaluate single infections and co-infections.

### Statistics

Isolation success rates between SC and TEv media were compared using McNemar’s test, as both tests were applied to the same individuals and the data were paired. Comparisons involving culture, cytology and cPCR were analysed using Fisher’s exact test (i.e. positive or negative) across all sampling months to assess prevalence and potential temporal variations in parasite detection. All statistical analyses were performed using R software (R Core Team, version 4.5.0). McNemar’s test was conducted using the mcnemar.test() function, and Fisher’s exact tests were conducted using the fisher.test() function.

### Monitoring of natural infection of Trypanosomatidae

To gain new insight into the course of natural infection, two geckos with a single infection with *L*. (*S*.) *tarentolae* or *T. platydactyli*, collected at the end of the sand fly season (October), were kept under room temperature in individual terrariums with controlled feeding and water supply for 4 months to monitor parasitaemia. Every 35 days, the animals underwent blood collection for cytology, cPCR for *T. platydactyli* and *L*. (*S*.) *tarentolae* and culturing in SC medium, following the procedures cited above. Additionally, the gecko infected with *L*. (*S*.) *tarentolae* was tested by real-time PCR (qPCR) using primers L.i.t. -ITS1-F 5′-GCAGTAAAAAAAAGGCCG-3′ and L.i.t. -ITS1-R 5′-CGGCTCACATAACGTGTCGCG-3′, and the hydrolysis TaqMan-MGB probe L.t. −6-FAM-5′-CACGCCGCGTATACAAAAACAC-3′-non-fluorescent quencher-minor groove binder(NFQ-MGB) (Applied Biosystems; Foster City, USA), targeting 150 bp of the internal transcribed spacer 1 (ITS1) region of *L*. (*S*.) *tarentolae*, as previously described [35]. Reactions were run in triplicate using a standard curve to quantify the amount of *L*. (*S*.) *tarentolae* DNA (Quantification cycle - Cq values). gDNA extracted from cultured promastigotes of the reference strain *L*. (*S*.) *tarentolae* LEM124 was used as a positive control; an NTC and DNA extracted from uninfected gecko blood were included in each run as negative controls. Serial dilutions were prepared to construct standard curves, ranging from 2.89×10⁵ to 2.89×10° copies per microlitre. qPCR reactions were carried out in a final volume of 20 µl, consisting of 10 µl of IQ Supermix (Bio-Rad Laboratories, USA), 7.22 µl of DEPC (diethylpyriarbinate)-treated, pyrogen-free, DNase/RNase-free water (Invitrogen, Carlsbad, CA, USA), 2 µl of template DNA, 950 nM of each primer and 200 nM of FAM TaqMan-MGB probes. The thermal cycling conditions consisted of a hot start at 95 °C for 3 min, and 40 cycles of denaturation (95 °C for 10 s) and annealing–extension (55 °C for 30 s). The qPCR was performed on a CFX96^TM^ Real-Time System (Bio-Rad Laboratories, Inc., USA), and the increase in fluorescent signals was registered during the extension step of the reaction and analysed using the CFX Manager^TM^ software, version 3.1 (Bio-Rad).

## Results

### Culture

*T. platydactyli* was successfully isolated from 13 *T*. *mauritanica* using SC medium (27%) and from 6 (12.5%) using TE medium (Table S2). Additionally, *L*. (*S*.) *tarentolae* was isolated from two geckos (i.e. 4.15%) exclusively with SC medium (Table S2). All blood samples from *P. siculus* and *H. turcicus* were negative in culture. Comparison between the two types of culture media demonstrated a statistically significant difference in parasite isolation rates, with SC yielding more positive results than TE (McNemar’s test, *P*=0.0156).

**Fig. 1.**
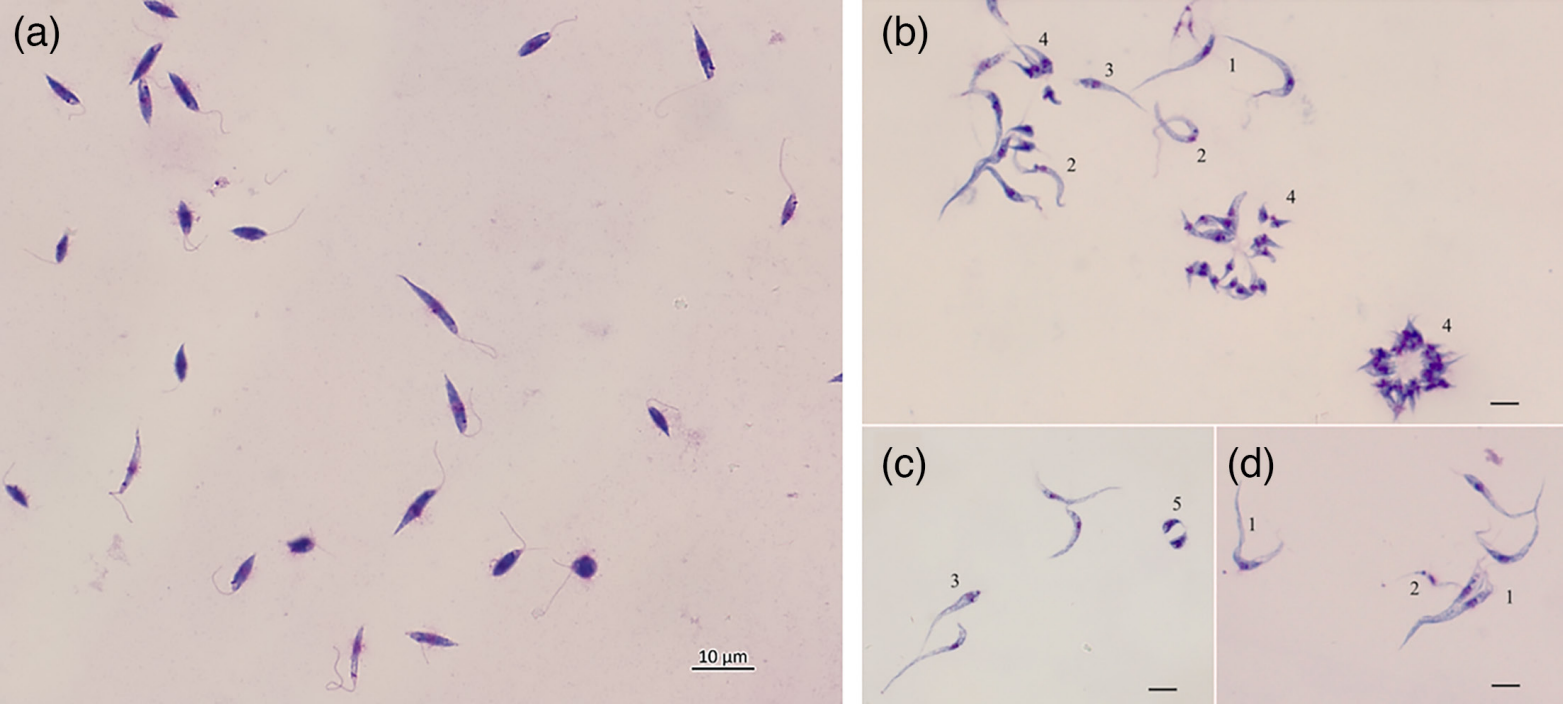
Trypanosomatids isolated from *T. mauritanica* blood in culture, stained with Diff-Quik and observed under a light microscope at 1,000× magnification using oil immersion. (**a**) *L.* (*S.*) *tarentolae* promastigotes; (**b**) *T. platydactyli* trypomastigotes (1 and 3), epimastigotes (2), metacyclic forms (4) and spheromastigotes (5).

*L.* (*S*.) *tarentolae* culture cells showed homogeneous, elongated promastigote morphology (Fig. 1a), with movements in a straight line, whereas *T. platydactyli* culture showed different morphological features (Fig. 1b–d), with different movement trajectories. The longer forms of *T. platydactyli* (Fig. 1b, d, number 1), similar to the trypomastigotes present in blood smears, exhibited slow and limited movements, not moving in a straight line like *Leishmania*, and occasionally spinning around themselves, with a visibly undulating membrane. Another form of *T. platydactyli* had a similar morphology to trypomastigote but was smaller in size (Fig. 1b, d, number 2), and exhibited more vigorous movements than the longer form, spinning around themselves, and sometimes moving in a straight line like *Leishmania*. The cytoplasm and nucleus of these forms stain more lightly compared with other forms, making it possible to clearly observe the separation between the nucleus and the kinetoplast. A third form of *T. platydactyli* presented a more piriform shape near the flagellum insertion, with the nucleus and kinetoplast positioned in the same region, with limited movement (Fig. 1b, c, number 3). In some microscopic fields, *T. platydactyli* formed circular parasite clusters, composed of very small structures of piriform shape, and the nucleus strongly stained purple (Fig. 1b, number 4). These forms exhibited movement primarily of the flagellum. Rarely, piriform stages were also observed, with the cytoplasm staining dark purple, preventing the visualization of the nucleus and kinetoplast, and exhibiting limited movement (Fig. 1c, number 5).

Out of the 12 dissected *S. minuta* that tested positive for flagellates, only one yielded an isolate of *T. platydactyli*, obtained in June. The culture from *S. minuta* displayed the same morphological features as that obtained from blood samples. In five cases, the gut contents contained dead trypanosomatids, and despite attempts, cultures could not be established. The remaining six cultures were contaminated with fungi and/or bacteria and were therefore discarded.

### Cytology

**Fig. 2.**
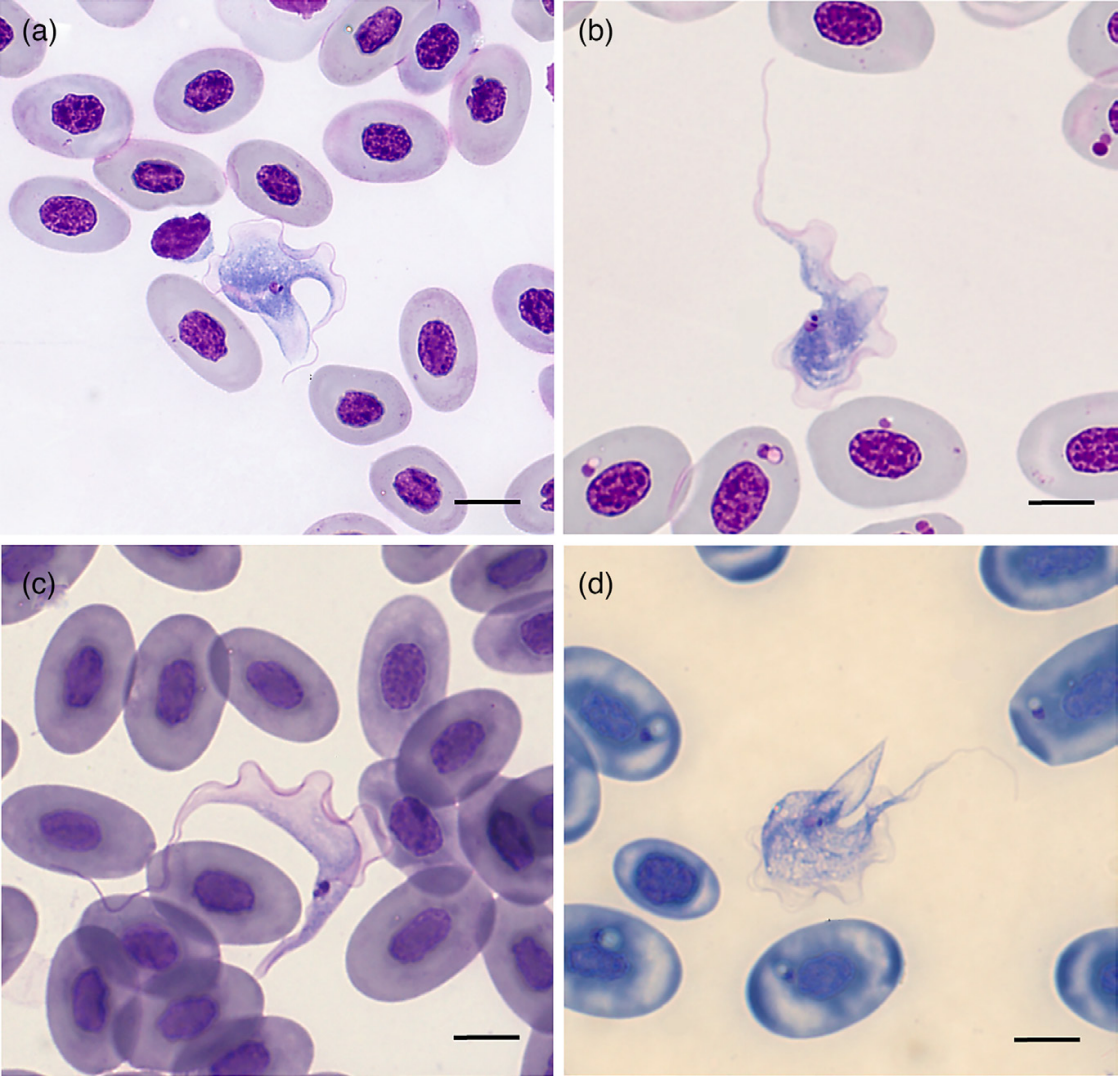
Trypomastigotes of *T. platydactyli* in geckos’ blood smear, stained with Diff-Quik and observed under a light microscope at 1,000× magnification using oil immersion.

Twelve (25%) *T. mauritanica* blood smears were positive for trypanosomatids on cytology, while all blood samples from *P. siculus* and *H. turcicus* were negative (Table S2). The positive smears contained variable numbers of extracellular trypomastigotes, ranging from one (*n*=7) up to seven (*n*=1). The trypomastigotes’ size was similar to or larger than that of erythrocytes, with a long and robust body that was often curved (mean length: 49.7±4.6 µm). Trypomastigotes exhibited a progressively tapering anterior end with the flagellum (mean length: 12.3±1.6 µm), while the posterior end ended in a very long, slender tip. The nucleus and kinetoplast were very close to each other and located at the posterior end of the body. The nucleus had an oval, relatively regular shape, was situated anterior to the kinetoplast and stained lilac with the Diff-Quick stain (Fig. 2). Measurements of trypomastigotes are reported in Table 1.

**Table 1.** Measures of 15 *T. platydactyli* extracellular trypomastigotes observed in *T. mauritanica* blood smears stained with Diff-Quik All measures are given in µm.

Trypomastigote no.	Total body length (including flagellum)	Flagellum	Width (including undulanting membrane)	Posterior end to nucleus	Anterior end to nucleus	Nucleus
**1**	46.9	12.97	10.76	13.64	20.29	1.94×1.84
**2**	51.88	12.47	10.34	12.02	27.39	1.84×2.15
**3**	48.78	12.89	8.08	12.07	23.82	1.56×1.34
**4**	44.73	10.6	7.94	11.51	22.62	1.90×1.80
**5**	49.60	12.43	9.22	12.56	24.61	1.54×1.33
**6**	41.56	8.64	12.15	11.13	21.79	1.47×1.38
**7**	51.08	12.9	9.84	12.83	25.35	1.72×1.33
**8**	51.4	13.23	8.04	10.3	27.87	–
**9**	49.91	12.69	8.24	13.78	23.44	1.79×1.66
**10**	56.21	16.24	9.5	14.39	25.58	1.87×1.80
**11**	49.77	10.55	11.95	12.12	27.1	1.86×1.42
**12**	51.86	11.73	8.26	16.36	23.77	1.52×1.55
**13**	60.17	12.95	7.64	13.02	34.2	1.56×1.26
**14**	47.8	11.5	7.78	12.47	23.83	2.05×1.92
**15**	44.24	13.58	8.37	8.89	21.77	2.13×1.89
**Mean**	49.7±4.6	12.3±1.6	9.2±1.5	12.5±1.7	24.9±3.4	–

– indicates measurements that could not be obtained for the evaluated parasite.

### Genome assembly, validation and annotation

**Fig. 3.**
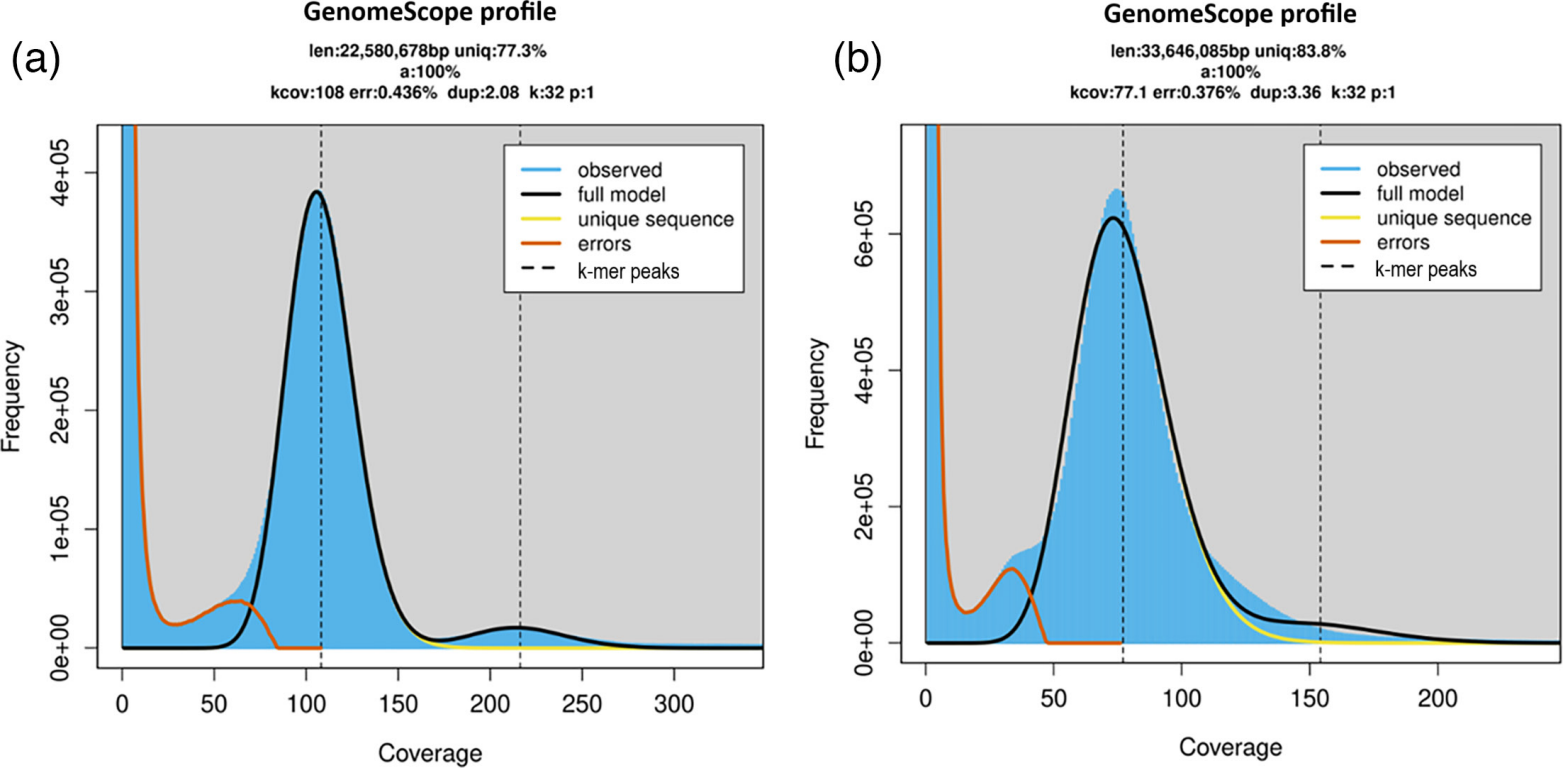
K-mer frequency distributions (k=32) modelled with GenomeScope 2.0 using a haploid model (*P*=1). The main peak corresponds to the nuclear genome coverage. (**a**) *T. platydactyl*i: estimated haploid genome size ≈22.58 Mb, average k-mer coverage 108×, 77.3% unique sequence, error 0.44%, duplication 2.08%. (**b**) *L.* (*S.*) *tarentolae*: estimated haploid genome size ≈33.65 Mb, average k-mer coverage 77×, 83.8% unique sequence, error 0.38%, duplication 3.36%. High-copy k-mers (>1,000×) were removed prior to modelling to reduce kinetoplast DNA influence. (len: estimated haploid genome size; uniq: unique sequence; a: indicates the fraction of the genome model successfully fit using a haploid assumption (ploidy = 1); kcov: average k-mer coverage; err: error; dup: duplication).

For *T. platydactyli*, the histogram of k-mer distribution exhibited a unimodal pattern, with no evidence of a secondary heterozygous peak (Fig. 3a). The haploid model (*P*=1) provided the best fit, yielding an estimated genome size of ~22.6 Mb, with 77.3% unique sequence, an error rate of 0.44% and a duplication rate of 2.1%. For the reference genome assembly, a total of 27,215,245 paired-end reads were processed, of which 26,627,375 (97.8%) successfully mapped to the *T. platydactyli* reference genome. Among them, 24,233,316 reads (89.8%) were properly paired, while 1.1% were singletons, with an average coverage of ~186×. After haploid variant calling and stringent filtering, 2,491 SNPs and 325 INDELs (Transition/Transversion Ti/Tv=2.10) were retained. During consensus generation, all 2,816 high-confidence variants were successfully applied to the reference genome, resulting in the final corrected assembly. Fig. S1 summarizes the main variant statistics, including depth distribution, transition/transversion ratios across quality thresholds and INDEL size distribution. Assembly metrics are provided in Table S3. BUSCO analysis revealed that the final assembly of *T. platydactyli* contained 95.1% complete and 4% missing BUSCO genes (i.e. 5,397 total) (Table S4). Overall, 1.86% of the *T. platydactyli* genome (i.e. 383,160 bp) was masked using the trypanosomatid-specific library. DIRS-like (*Dictyostelium* intermediate repeat sequence) retrotransposons represented the most abundant fraction, followed by Long Interspersed Nuclear Elements (LINE) and LTR (Long Terminal Repeat)/VIPER (Vestigial Interposed Retroelement) (Table S5).

For *L*. (*S*.) *tarentolae*, the k-mer distribution also showed a unimodal profile, lacking a detectable heterozygous peak (Fig. 3b). The haploid model (*P*=1) estimated a genome size of 33.6 Mb, with 83.8% unique sequence, an error rate of 0.38% and a duplication rate of 3.4%. For the reference assembly, a total of 18,556,608 paired-end reads were processed, of which 17,721,645 (95.2%) successfully mapped to the reference genome. Among them, 17,480,012 reads (94.2%) were properly paired, while 0.05% were singletons, with an average coverage of ~82×. After stringent filtering, 17,210 SNPs and 3,180 INDELs were retained, with a Ti/Tv ratio of 1.71, indicating a high-quality variant set. Fig. S2 summarizes the main variant statistics, including depth distribution, transition/transversion ratios across quality thresholds and INDEL size distribution. During consensus generation, 20,328 variants were successfully applied to the reference genome. Assembly metrics are provided in Table S3. BUSCO analysis revealed that the final assembly of *L*. (*S.*) *tarentolae* contained 98.3% complete and 1.6% missing BUSCO genes (i.e. 6,640 total) (Table S4). Overall, 2.69% of the *L*. (*S.*) *tarentolae* genome (i.e. 858,982 bp) was masked using the trypanosomatid-specific library. LINE retrotransposons represented the most abundant fraction, with only trace amounts of DIRS-like elements (Table S5).

**Fig. 4.**
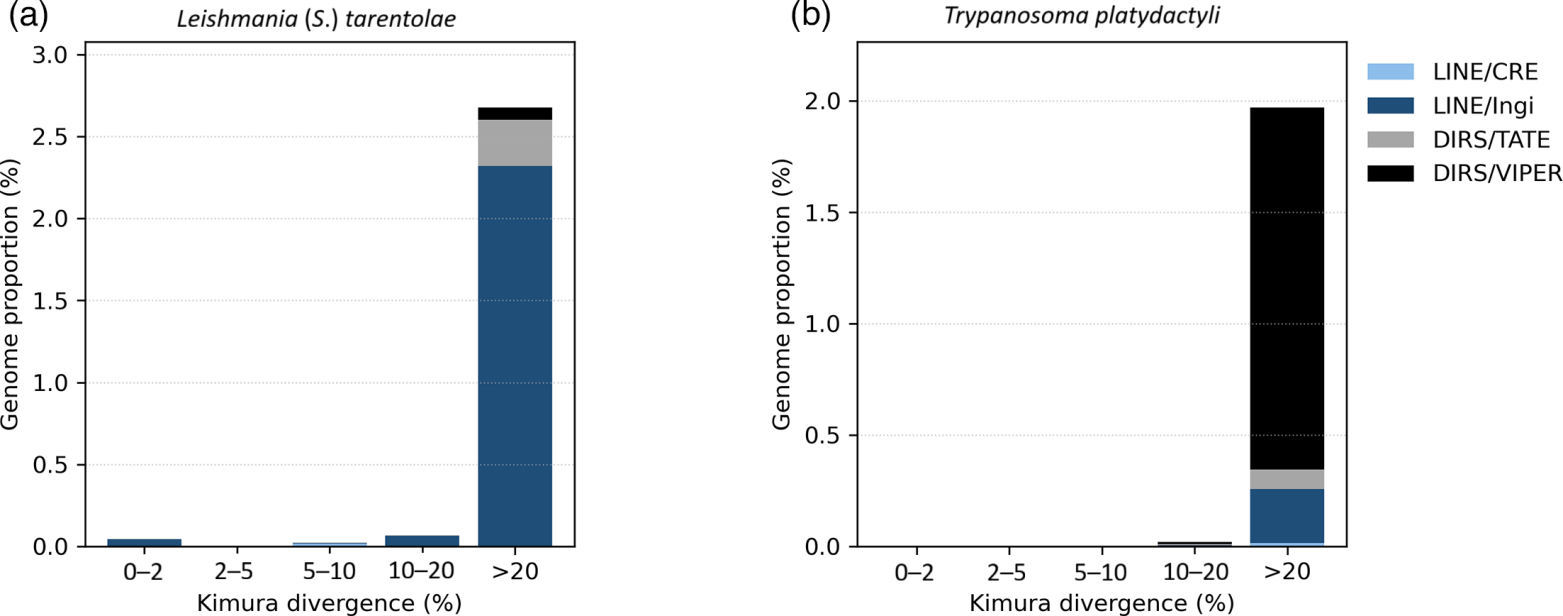
Repeat landscapes of *L. (S.) tarentolae* (**a**) and *T. platydactyli* (**b**). Genomic proportion of TE classes across K2P divergence bins. Low-divergence copies (≤5%) indicate recent insertions, whereas highly diverged elements (>20%) represent ancient or inactive copies. CRE: Crithidia retrotransposable element.

 K2P divergence profiles revealed distinct TE landscapes between the two species (Fig. 4). In *L*. (*S*.) *tarentolae* (Fig. 4a), LINE/Ingi elements dominate the repetitive fraction and show a small but clear accumulation of low-divergence copies (≤5–10%), consistent with relatively recent insertions; DIRS elements - TATE (Telomerase-Associated Transposable Element)/VIPER occur at much lower proportions. By contrast, *T. platydactyli* (Fig. 4b) has a lower overall repeat load and is dominated by DIRS/VIPER elements, with no enrichment of recent insertions. Although the most recently diverged copies in *L*. (*S*.) *tarentolae* represent <0.05% of the genome, it contrasts with the flat landscape of *T. platydactyli* and points to residual, lineage-specific transpositional activity in *Leishmania*.

### Molecular biology

Twenty-two out of 48 *T*. *mauritanica* samples (45.8%) tested positive by cPCR targeting the *β-tubulin* gene (Fig. S3). Among these, nine samples (18.75%) were positive only for *T. platydactyli*, six (12.5%) only for *L*. (*S*.) *tarentolae* and seven (14.6%) were co-infected (Table S2). All *P. siculus* and *H. turcicus* samples were negative in *β-tubulin* cPCR for both trypanosomatids. The LOD for the new cPCR was successfully determined: all three replicates of *T. platydactyli* (using primers F and R1) and *L*. (*S*.) *tarentolae* (using primers F and R2) amplified consistently up to 1 pg µl^−1^ dilution, confirming the sensitivity of the assay. The cPCR using three primers to detect single and co-infections did not interfere with the LOD.

Out of 208 *S*. *minuta* sand flies tested, 19 (9.1%) were positive for *T. platydactyli*, 30 (14.4%) for *L*. (*S.*) *tarentolae* and 15 (7.2%) were co-infected with both. The prevalence of *T. platydactyli* infection in gecko blood samples varied slightly between months, reaching 28.6% in May, 25.0% in June, 37.5% in July and 20.0% in both September and October. *L.* (*S*.) *tarentolae* prevalence was lower in May (14.3%) but increased to 50.0% in both June and July, before decreasing to 20.0% in September and October. Statistical analysis indicated no significant difference in the prevalence of flagellates between months (*P*>0.05).

### Monitoring the natural infection

Of the two geckos naturally infected and monitored over 4 months, the first, infected with *L*. (*S*.) *tarentolae*, tested consistently positive by cPCR, but negative on cytology and culture (except for the initial time point, which yielded a positive culture after 30 days of inoculation).

The second gecko, infected with *T. platydactyli*, tested positive both by cytology and cPCR, with cultures becoming positive after 10 days of inoculation. Notably, cPCR positivity and the detection of trypomastigotes by cytology were observed only during the first (October) and final sampling (February). Despite these intermittent PCR and cytology results, cultures remained positive throughout the 4 months, indicating parasite persistence at low parasitaemia levels (Table 2).

**Table 2.** Monitoring of natural infection in geckos infected with single infection by *T. platydactyli* or *L.* (*S.*) *tarentolae*, assessed through cytology, dqPCR, cPCR (*β-tubulin*) and culture at different time points

					Culture		
**Sampling**	**Infection**	**Cytology**	dqPCR ITS1 (mean Cq^*^)	**cPCR *β*-*tubulin***	**10** days	**20** days	**30** days
**1**	***L. tarentolae***	Negative	1.09×10^2^	+	Negative	Negative	Positive
	***T. platydactyli***	Positive	Negative	+	Positive	Positive	Positive
**2**	***L. tarentolae***	Negative	2.76×10^1^	+	Negative	Negative	Negative
	***T. platydactyli***	Negative	Negative	−	Negative	Positive	Positive
**3**	***L. tarentolae***	Negative	2.05×10^2^	+	Negative	Negative	Negative
	***T. platydactyli***	Negative	Negative	−	Positive	Positive	Positive
**4**	***L. tarentolae***	Negative	2.23×10^1^	+	Negative	Negative	Negative
	***T. platydactyli***	Positive	Negative	+	Positive	Positive	Positive

*Mean copy per microlitre quantification values.

dqPCR, duplex real-time PCR.

## Discussion

In this study, a new cPCR protocol for diagnosing single and co-infections by *L*. (*S*.) *tarentolae* and *T. platydactyli* in reptiles and *S. minuta* sand flies was successfully developed. In addition, protocols and data on the isolation of new strains of both trypanosomatids are provided, along with morphological features of *T. platydactyli*. Although *L*. (*S*.) *tarentolae* was more frequently detected in June and July, statistical analysis (i.e. Fisher’s exact test) did not confirm a significant difference over time, possibly due to sample size limitations. In contrast, *T. platydactyli* was consistently detected throughout the entire sampling period.

Even though *L*. (*S*.) *tarentolae* was previously isolated in TE medium [8], the data presented herein suggest that SC medium is more effective in supporting trypanosomatid growth. Indeed, *T. platydactyli* was better isolated in SC medium (27%) compared with TE medium (12.5%), whereas *L*. (*S*.) *tarentolae* was isolated only in SC. This could be due to the fact that SC might be more suitable for the isolation of both trypanosomatids, as it has a different composition that may provide the differential nutrients required for their optimal growth. Nonetheless, the choice of media could conflate the true prevalence of *L*. (*S*.) *tarentolae* with isolation performance, particularly considering that a high number of animals tested positive by molecular methods, while only two isolates were successfully obtained. Notably, the culture of *Leishmania* spp. is challenging due to their nutritional requirements and susceptibility to contamination, which can further reduce isolation rates *in vitro* [36,37]. The case of *L*. (*S*.) *tarentolae* is even more problematic, as little is known about the species’ biology, and consequently its precise nutritional and physiological requirements (e.g. optimal temperature, serum dependence) remain poorly defined. These knowledge gaps likely contribute to the difficulty of establishing *L*. (*S*.) *tarentolae* in culture and may partly explain the low isolation success despite evidence of circulation in host populations. Further comparative studies, including the use of other culture media, growth curve analyses and different serum supplementation strategies, could help clarify the specific nutritional and physiological requirements of these protozoa in culture.

Data also indicate that the gecko *T. mauritanica* is the most common reptilian host maintaining the trypanosomatid cycle in southern Italy. Although *L. infantum* and *L*. (*S*.) *tarentolae* have previously been detected molecularly in blood and tissues from the lizard *P. siculus*, neither *L*. (*S*.) *tarentolae* nor *T. platydactyli* have ever been isolated from this species [8]. Coherently, monitoring natural infections in geckos has revealed distinct infection dynamics between these trypanosomatids. *L.* (*S*.) *tarentolae* was not detected by cytology and was rarely detected by culture, while *T. platydactyli* exhibited persistent culture positivity even when cPCR results were transiently negative. The variation in *L*. (*S*.) *tarentolae* parasitaemia may suggest that: (i) the circulating protozoan periodically localizes in host tissues (e.g. spleen, bone marrow), for example, during the intracellular amastigote stage, and/or (ii) an immune-mediated clearance periodically occurs. This pattern aligns with previous reports of the intracellular and tissue tropism of *Leishmania* spp. [38,39] and highlights the challenges in detecting this parasite using cytological and culture-based methods alone. Conversely, *T. platydactyli* exhibited a more stable presence in the bloodstream, with consistent isolation in culture across multiple time points. This could be due to its extracellular development and/or to its capacity to evade the host immune response. These findings reinforce the importance of combining multiple diagnostic techniques (i.e. molecular, cytological and culture-based methods) to improve the detection of trypanosomatids in reptile hosts.

The evidence of co-infections by *L*. (*S*.) *tarentolae* and *T. platydactyli* in geckos and *S. minuta* sand flies suggests that both the vertebrate and the vector provide entozoic niches, which may facilitate the co-existence of these trypanosomatids. Such co-infections raise the intriguing possibility of direct or indirect interactions between these parasites within the vector or host. For instance, their simultaneous occurrence in the gut of sand flies or in reptilian blood may foster competitive or cooperative interactions that could affect parasite survival and transmission, while also providing opportunities for genetic interactions. Indeed, hybrid formation between *Leishmania major* and *L. infantum* within the vector’s gut has been demonstrated [40]. Notably, reports of hybridization events at the subgenus level (e.g. between *Leishmania* and *Sauroleishmania*) highlight the potential for genomic compatibility and transcriptomic adaptation, even between distantly related species [41]. Although direct evidence of gene exchange between *L*. (*S*.) *tarentolae* and *T. platydactyli* is lacking, the genomic plasticity of trypanosomatids suggests that they may be subjected to adaptive interactions, genome remodelling or even hybridization.

The absence of bimodality in the k-mer spectra of both *T. platydactyli* and *L*. (*S*.) *tarentolae* indicates extremely low heterozygosity, consistent with other trypanosomatids dominated by clonality or loss of heterozygosity [42]. Similar patterns have been documented in other non-pathogenic trypanosomatids, such as *T. melophagium* and *T. theileri*, which also exhibit reduced heterozygosity and compact genome sizes [25]. Such unimodal profiles may result from founder effects, narrow host/vector niches or the absence of a sexual cycle causing allelic drift [25]. To date, *T. platydactyli* has been exclusively detected in geckos of the genus *Tarentola* and in *Sergentomyia* phlebotomine sand flies in the Mediterranean basin [9,17]. For *L*. (*S*.) *tarentolae*, although molecular detection has extended to other hosts (e.g. dogs, humans) [7,9], successful isolations remain limited to geckos, reinforcing the idea of a restricted ecological niche. This extremely restricted host association, combined with a limited Mediterranean distribution, may have facilitated successive bottlenecks, further reducing allelic diversity. These host/vector constraints likely contribute to the unimodal k-mer spectra. Interestingly, the genome size predicted by GenomeScope (~22–23 Mb) was slightly larger than the short-read assembly (i.e. 20.55 Mb), which matches the current reference genome (GCA_030849675.1). This discrepancy likely reflects the tendency of k-mer-based approaches to capture repetitive content and low-coverage regions that are often collapsed or lost in assemblies derived exclusively from short reads [43]. Thus, GenomeScope provides a complementary view that may approximate the true genome size more closely than short-read assemblies alone.

The contrasting repeat landscapes observed between *L*. (*S*.) *tarentolae* and *T. platydactyli* likely reflect differences in their genomic regulation and evolutionary histories. The low-divergence LINE-like copies detected in *L*. (*S*.) *tarentolae* suggest that, although transposon activity is limited, this species may have retained residual or episodic mobilization potential, possibly associated with stress-induced genome remodelling or adaptation to reptilian hosts [44,46]. In contrast, the absence of low-divergence copies and the dominance of highly degraded DIRS-like elements in *T. platydactyli* indicate long-term silencing and loss of TE activity [45]. Such quiescence could result from stronger genome stability constraints in the latter, where maintenance of a compact, stable genome may be advantageous for survival in a narrow host–vector system [47]. These contrasting dynamics imply that even within closely related reptile-associated trypanosomatids, the evolutionary trajectories of TEs can diverge substantially, reflecting lineage-specific ecological and cellular contexts [48].

In contrast to the previously reported absence of TATE elements in the *L*. (*S*.) *tarentolae* strain [33], our results revealed a small but consistent fraction of DIRS/TATE-like sequences. This discrepancy likely stems from the different genome assemblies used for masking. While [33] employed the earlier long-read PacBio assembly (GCA_009731335.1), which lacks chromosome-level contiguity and underrepresents repetitive regions, the present analysis was based on the updated assembly (GCA_033953505.1), with improved coverage of repetitive and subtelomeric loci. The enhanced assembly quality likely allowed the recovery of residual TATE-like elements that were collapsed or absent in the earlier genome. Nevertheless, because the present analyses relied on reference-guided strategies, no *de novo* TEs were identified. Our findings should therefore be interpreted as an updated masking of existing TEs.

Variant calling of *L*. (*S*.) *tarentolae* yielded an order of magnitude more SNPs and small indels than *T. platydactyli*. The transition/transversion ratio exceeded 3 among high-quality sites and declined gradually as lower confidence variants were included, consistent with genuine sequence divergence rather than technical noise. The contrast was consistent with reference relatedness: the *T. platydactyli* reference derives from an Italian isolate from the same region as the present sample, whereas the *L*. (*S*.) *tarentolae* reference genome (GCA_033953505.1) originates from Algeria. Accordingly, the *Trypanosoma* comparisons reflect within-region variation, whereas greater pairwise divergence is expected for *L*. (*S*.) *tarentolae* in the present case, consistent with the geographic structuring documented for several *Leishmania* species across Old and New World regions [49,51]. Although long-term laboratory culture has been associated with shifts in repetitive content [52], geographic separation likely explains most of the observed SNPs.

The recent development of curated, lineage-specific repeat libraries for trypanosomatids (e.g. [33]) and new quality assemblies for reptile trypanosomatids (i.e. GCA_033953505.1) represents a major step forward in accurately characterizing these genomes. Nevertheless, continued refinement and periodic updates of these resources remain essential, as ongoing genome sequencing of diverse isolates will likely uncover additional lineage-restricted elements. Expanding and maintaining such databases will enhance the precision of repeat annotation and enable more robust evolutionary comparisons across kinetoplastid species. In parallel, future studies employing long-read and hybrid sequencing technologies [e.g. single-molecule real-time (SMRT, PacBio) or Oxford Nanopore] will be crucial to fully resolve complex and repetitive genomic regions, particularly in poorly studied non-pathogenic trypanosomatids such as *T. platydactyli*, for which long-read data are still unavailable. Such efforts will provide deeper insights into genome plasticity, adaptation and host–parasite interactions in these understudied lineages.

Altogether, this multidisciplinary framework, integrating genomic, phenotypic and ecological evidence, provides new insights into reptile-associated trypanosomatids.

## Supplementary material

10.1099/mgen.0.001567 Uncited Supplementary Material 1.

## Abbreviations

cPCR: conventional PCR
DIRS: dictyostelium intermediate repeat sequence
DP4: read depth per allele per strand
gDNA: genomic DNA
INDEL: insertion-deletion
ITS1: internal transcribed spacer 1
K2P: Kimura 2-parameter
LINE: long interspersed nuclear elements
LOD: limit of detection
MGB: minor groove binder
MQ0F: mapping quality zero fraction
NTC: no-template control
qPCR: real time quantitative PCR
QUAL: Phred-scaled quality score
TATE: telomerase-associated transposable element
Ti/Tv: transition/transversion
VDB: variant distance bias
