# Supplementary material for: Epidemiological dynamics of Leishmania (Sauroleishmania) tarentolae and Trypanosoma platydactyli in reptile hosts and sand flies: from isolation to genome assembly
Source: Microb Genom. 2025 Dec 9;11(12):001567. doi: 10.1099/mgen.0.001567 (PMC12688035; doi:10.1099/mgen.0.001567)
Supplement: Uncited Supplementary Material 1. [file mgen-11-01567-s001.pdf]

Epidemiological dynamics of *Leishmania (Sauroleishmania) tarentolae* and *Trypanosoma platydactyli* in reptile hosts and sand flies: from isolation to genome assembly

Supplementary Tables

Table S1. Primers designed for *β-tubulin* gene for differentiation of *Leishmania tarentolae*/*Leishmania infantum* and *Trypanosoma platydactyli*, with fragment size (bp) and thermocycling conditions.

	Trypanosoma platydactyli	***Leishmania tarentolae* and *Leishmania infantum***	***Trypanosoma platydactyli*, *Leishmania tarentolae* and *Leishmania infantum***
**Primer F**	5’ – TCCGCCCRGACAACTTCATC – 3’	5’ – TCCGCCCRGACAACTTCATC – 3’	5’ – TCCGCCCRGACAACTTCATC – 3’
**Primer R1**	5’ – AACGGTGTCCGACACGCGG – 3’	-	5’ – AACGGTGTCCGACACGCGG – 3’
**Primer R2**	-	5’ – GATCCACTCAATGAAGTAGGAC - 3’	5’ – GATCCACTCAATGAAGTAGGAC -3’
**Amplicon size (includind primers)**	763bp	269bp	763bp for *T. platydactyli*<br>269bp for *Leishmania tarentolae* and *Leishmania infantum*
**Thermocycling conditions**	Hot-start at 95 °C for 10 min, and 35 cycles of denaturation at 95 °C for 15 s, annealing at 62ºC for 40 s, 72ºC for 1 min; and a final extension at 72ºC for 7 min	Hot-start at 95 °C for 10 min, and 35 cycles of denaturation at 95 °C for 15 s, annealing at 62ºC for 30 s, 72ºC for 30 s; and a final extension at 72ºC for 7 min	Hot-start at 95 °C for 10 min, and 35 cycles of denaturation at 95 °C for 15 s, annealing at 62ºC for 40 s, 72ºC for 1 min; and a final extension at 72ºC for 7 min

Table S2. Trypanosomatid detection in reptiles *Tarentola mauritanica*, *Podarcis siculus* and *Hemidactylus turcicus* using isolation in Tobie Evans (TE) or Schneider (SC) medium, blood cytology, and conventional PCR targeting *β-tubulin* gene fragment for diagnosis of *Trypanosoma platydactyli* and *Leishmania* (*Sauroleishmania*) *tarentolae*.

ID number	Species	Collection date	Sex	Age	Culture		Blood cytology	dqPCR (Mean Cq*)	cPCR ***β-tubulin***
					SC	TE			
**1**	*Tarentola mauritanica*	10 May	F[1]	A[3]	Negative	Negative	Negative	Negative	Negative
**2**	*Tarentola mauritanica*	10 May	M[2]	SA[4]	Negative	Negative	Negative	Negative	Negative
**3**	*Tarentola mauritanica*	10 May	M	SA	Negative	Negative	Negative	Negative	Negative
**4**	*Tarentola mauritanica*	10 May	F	SA	Negative	Negative	Negative	Negative	Negative
**5**	*Tarentola mauritanica*	10 May	F	A	*Trypanosoma* sp.	*Trypanosoma* sp.	Negative	Negative	Negative
**6**	*Tarentola mauritanica*	10 May	M	A	Negative	Negative	Negative	Negative	Negative
**7**	*Tarentola mauritanica*	10 May	F	SA	Negative	Negative	Negative	Negative	Negative
**8**	*Tarentola mauritanica*	10 May	F	SA	Negative	Negative	Negative	*L. tarentolae*<br>2.68×10^2	*T. platydactyli*<br>*L. tarentolae*
**9**	*Tarentola mauritanica*	10 May	F	SA	*Trypanosoma* sp.	Negative	Negative	Negative	Negative
**10**	*Tarentola mauritanica*	10 May	F	SA	Negative	Negative	Trypomastigote	Negative	Negative
**11**	*Podarcis siculus*	10 May	M	A	Negative	Negative	Negative	Negative	Negative
**12**	*Podarcis siculus*	10 May	M	A	Negative	Negative	Negative	Negative	Negative
**13**	*Tarentola mauritanica*	16 May	M	A	*Trypanosoma* sp.	*Trypanosoma* sp.	Trypomastigote	Negative	Negative
**14**	*Tarentola mauritanica*	16 May	M	A	Negative	Negative	Negative	Negative	Negative
**15**	*Tarentola mauritanica*	16 May	F	SA	Negative	Negative	Negative	Negative	Negative
**16**	*Tarentola mauritanica*	16 May	M	A	Negative	Negative	Negative	Negative	Negative
**17**	*Tarentola mauritanica*	16 May	F	A	Negative	Negative	Negative	Negative	*T. platydactyli*
**18**	*Tarentola mauritanica*	16 May	M	A	*Trypanosoma* sp.	Negative	Trypomastigote	Negative	Negative
**19**	*Tarentola mauritanica*	16 May	M	SA	Negative	Negative	Negative	Negative	*T. platydactyli*
**20**	*Tarentola mauritanica*	16 May	M	A	Negative	Negative	Negative	Negative	*T. platydactyli*<br>*L. tarentolae*
**21**	*Tarentola mauritanica*	16 May	M	SA	Negative	Negative	Negative	Negative	*T. platydactyli*<br>*L. tarentolae*
**22**	*Tarentola mauritanica*	16 May	F	SA	*Trypanosoma* sp.	Negative	Negative	Negative	Negative
**23**	*Tarentola mauritanica*	16 May	F	SA	*Trypanosoma* sp.	Negative	Negative	Negative	Negative
**24**	*Podarcis siculus*	16 May	F	SA	Negative	Negative	Negative	Negative	Negative
**25**	*Podarcis siculus*	16 May	F	A	Negative	Negative	Negative	Negative	Negative
**26**	*Tarentola mauritanica*	27 June	M	A	Negative	Negative	Negative	Negative	*L. tarentolae*

**27**	*Tarentola mauritanica*	27 June	F	A	*Trypanosoma* sp.	*Trypanosoma* sp.	Negative	Negative	*T. platydactyli*
**28**	*Tarentola mauritanica*	27 June	M	A	Negative	Negative	Trypomastigote	Negative	Negative
**29**	*Tarentola mauritanica*	27 June	F	A	*Trypanosoma* sp.	*Trypanosoma* sp.	Trypomastigote	Negative	*T. platydactyli*
**30**	*Tarentola mauritanica*	27 June	M	A	Negative	Negative	Negative	Negative	Negative
**31**	*Tarentola mauritanica*	27 June	M	A	Negative	Negative	Negative	Negative	*L. tarentolae*
**32**	*Tarentola mauritanica*	28 June	F	A	Negative	Negative	Negative	Negative	*T. platydactyli* *L. tarentolae*
**33**	*Tarentola mauritanica*	28 June	F	A	Negative	Negative	Trypomastigote	*L. tarentolae* $1.16x10^2$	*T. platydactyli* *L. tarentolae*
**34**	*Tarentola mauritanica*	18 July	F	SA	Negative	Negative	Negative	Negative	*T. platydactyli*
**35**	*Tarentola mauritanica*	18 July	F	SA	Negative	Negative	Negative	*L. tarentolae* $5.16x10^1$	*T. platydactyli* *L. tarentolae*
**36**	*Podarcis siculus*	18 July	M	A	Negative	Negative	Negative	Negative	Negative
**37**	*Tarentola mauritanica*	31 July	M	SA	Negative	Negative	Negative	*L. tarentolae* $2.01x10^4$	*T. platydactyli* *L. tarentolae*
**38**	*Tarentola mauritanica*	31 July	F	SA	Negative	Negative	Negative	Negative	Negative
**39**	*Tarentola mauritanica*	31 July	F	A	*Trypanosoma* sp.	Negative	Trypomastigote	Negative	*L. tarentolae*
**40**	*Tarentola mauritanica*	31 July	M	A	*Trypanosoma* sp.	*Trypanosoma* sp.	Trypomastigote	Negative	*T. platydactyli*
**41**	*Tarentola mauritanica*	31 July	F	A	*Trypanosoma* sp.	*Trypanosoma* sp.	Trypomastigote	Negative	*T. platydactyli*
**42**	*Tarentola mauritanica*	31 July	F	A	Negative	Negative	Negative	Negative	*L. tarentolae*
**43**	*Tarentola mauritanica*	31 July	F	SA	Negative	Negative	Negative	Negative	Negative
**44**	*Hemidactylus turcicus*	16 September	F	A	Negative	Negative	Negative	Negative	Negative
**45**	*Tarentola mauritanica*	16 September	M	A	Negative	Negative	Trypomastigote	Negative	Negative
**46**	*Tarentola mauritanica*	16 September	M	A	*Trypanosoma* sp.	Negative	Trypomastigote	Negative	*T. platydactyli*
**47**	*Tarentola mauritanica*	16 September	F	SA	Negative	Negative	Negative	Negative	Negative
**48**	*Tarentola mauritanica*	16 September	F	SA	*L. tarentolae*	Negative	Negative	*L. tarentolae* $9.51x10^4$	*L. tarentolae*
**49**	*Tarentola mauritanica*	16 September	F	SA	Negative	Negative	Negative	Negative	Negative
**50**	*Tarentola mauritanica*	18 October	F	A	Negative	Negative	Negative	Negative	Negative
**51**	*Podarcis siculus*	18 October	M	A	Negative	Negative	Negative	Negative	Negative

**52**	*Tarentola mauritanica*	18 October	F	A	*L. tarentolae*	Negative	Negative	*L. tarentolae* $1.09x10^2$	*L. tarentolae*
**53**	*Tarentola mauritanica*	18 October	M	A	*Trypanosoma* sp.	Negative	Trypomastigote	Negative	*T. platydactyli*
**54**	*Tarentola mauritanica*	18 October	F	SA	Negative	Negative	Negative	Negative	Negative
**55**	*Tarentola mauritanica*	18 October	M	A	Negative	Negative	Negative	Negative	Negative

Notes: [1] Female; [2] Male; [3] Adult; [4] Sub adult.

Table S3. Summary statistics of the corrected reference-guided assemblies of *Trypanosoma platydactyli* isolate G9 and *Leishmania (Sauroleishmania) tarentolae* isolate T33.

Metrics	*Trypanosoma platydactyli*	*Leishmania (S) tarentolae*
**# contigs (>= 0 bp)**	916	37
**# contigs (>= 1000 bp)**	737	37
**# contigs (>= 5000 bp)**	355	37
**# contigs (>= 10000 bp)**	307	37
**# contigs (>= 25000 bp)**	222	37
**# contigs (>= 50000 bp)**	134	36
**Total length (>= 0 bp)**	20548494	31929425
**Total length (>= 1000 bp)**	20424011	31929425
**Total length (>= 5000 bp)**	19672602	31929425
**Total length (>= 10000 bp)**	19325812	31929425
**Total length (>= 25000 bp)**	17807378	31929425
**Total length (>= 50000 bp)**	14660112	31900690
**Largest contig**	410903	2670596
**Total length**	20548494	31929425
**GC (%)**	48.82	57.06
**N50**	85176	1064421
**N90**	20838	549026
**auN**	111499.1	1177429
**L50**	69	11
**L90**	253	29
**# N's per 100 kbp**	19.91	42.78

Table S4. BUSCO completeness assessment of the *Trypanosoma platydactyli* and *Leishmania (Sauroleishmania) tarentolae* assemblies. Genome completeness was evaluated using BUSCO v5.8.2 in genome mode, with lineage-specific datasets from OrthoDB v12. The *Trypanosoma platydactyli* assembly was assessed using the trypanosoma_odb12 dataset (5,397 single-copy orthologs), and the *Leishmania Sauroleishmania tarentolae* assembly was evaluated with the leishmaniinae_odb12 dataset (6,640 orthologs).

	Trypanosoma platydactyli	*Leishmania (S) tarentolae*
**Complete BUSCOs**	5,135 (95.14%)	6,527 (98.3%)
**Complete and single-copy BUSCOs**	5,131 (95.1%)	6,501 (97.9%)
**Complete and duplicated BUSCOs**	4 (0.1%)	26 (0.4%)
**Fragmented BUSCOs**	47 (0.9%)	8 (0.1%)
**Missing BUSCOs**	215 (4%)	105 (1.6%)
**Total BUSCO groups searched**	5,397 (100%)	6,640 (100)

Table S5. Repeat landscape of the *Trypanosoma platydactyli* and *Leishmania (Sauroleishmania) tarentolae* genomes based on RepeatMasker analysis using the lineage-specific library TEs_trypanosomatids. Classes and families of repetitive elements were identified and soft-masked with RepeatMasker v4.1.6. The table reports the total length (bp) and proportion (%) of each repeat category relative to the genome assembly.

	Trypanosoma platydactyli		***Leishmania (Sauroleishmania) tarentolae*** **Total genome size**	
**Total genome size**	20,548,494		31,929,425	
**Masked bases bp (%)**	383,160 (1.86%)		858,982 (2.69%)	
**Class/Family**	**Length (bp)**	**Genome proportion (%)***	**Length (bp)**	**Genome proportion (%)***
**DIRS/VIPER**	312,205	1.52	23,671	0.07
**DIRS/TATE**	18,604	0.09	91,956	0.28
Total DIRS-like	*330,809*	*1.61*	*115,627*	*0.36*
**LINE/Ingi**	49,086	0.23	738,715	2.31
**LINE/CRE**	2,882	0.01	4,662	0.01
**LINE/I**	465	0.002	0	0
Total LINE-like	*52,433*	*0.25*	*743,377*	*2.33*

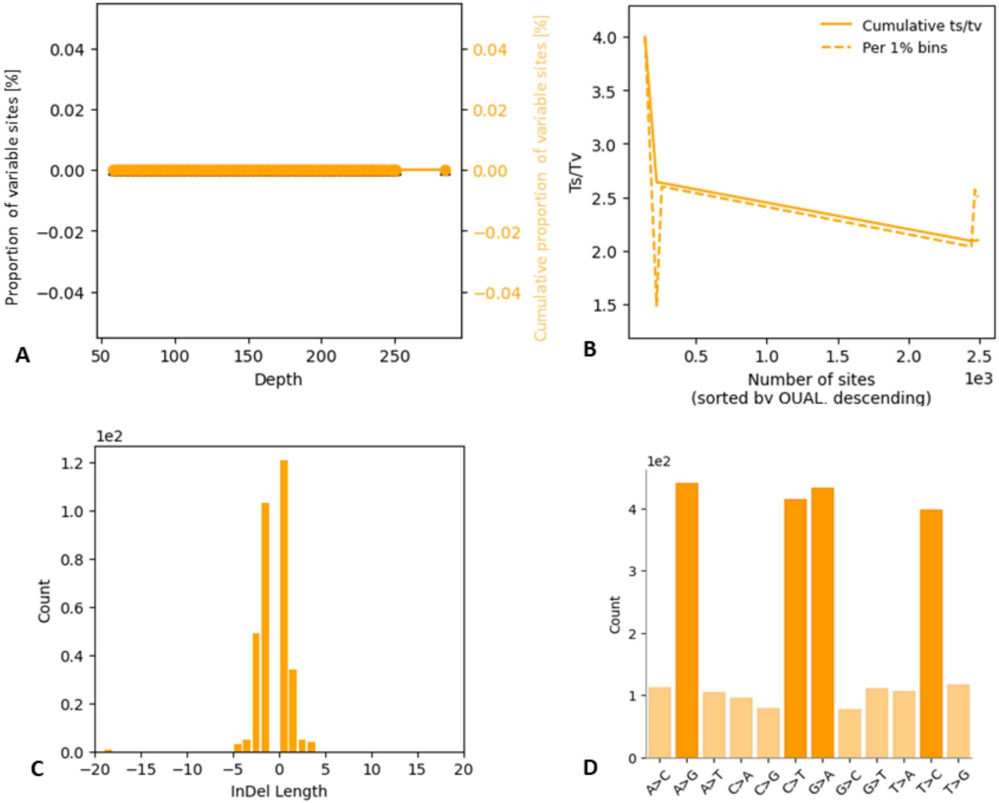

Supplementary Figure S1. Variant quality control and characterization in *Trypanosoma platydactyli*.

(A) Depth distribution across variant sites.

(B) Transition/transversion (Ti/Tv) ratio stratified by variant quality scores.

(C) Length distribution of INDELs.

(D) Spectrum of nucleotide substitutions.

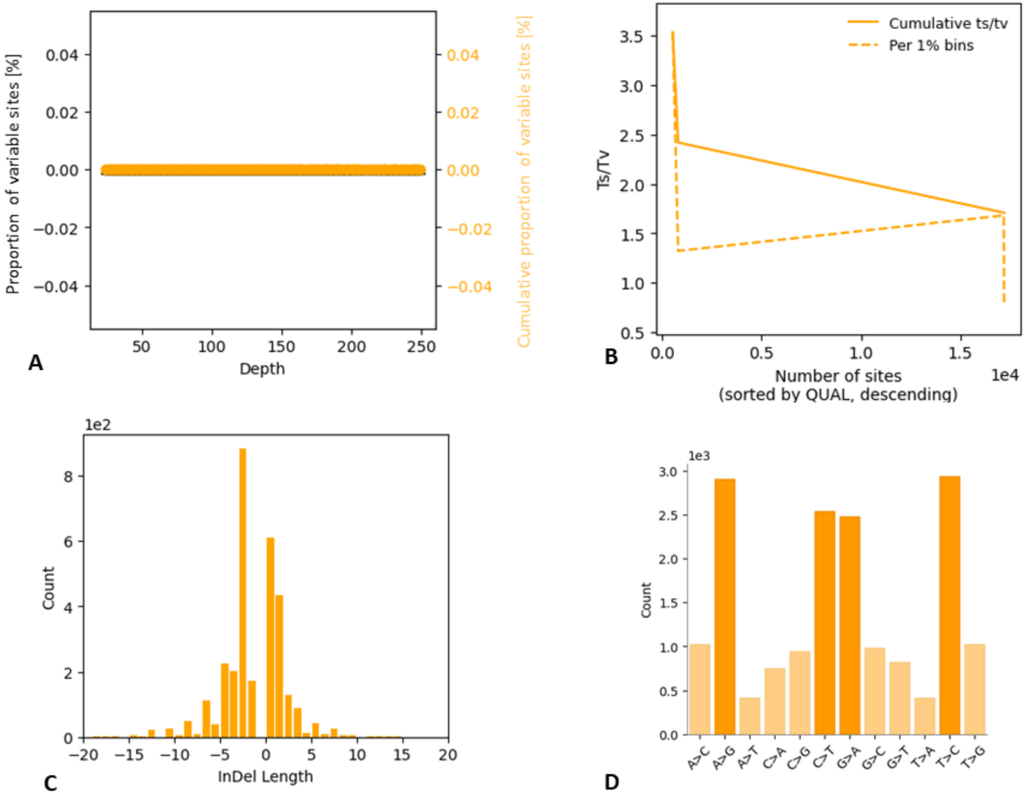

Supplementary Figure S2. Variant quality control and characterization in *Leishmania* (*Sauroleishmania*) *tarentolae*.

(A) Depth distribution across variant sites.

(B) Transition/transversion (Ti/Tv) ratio stratified by variant quality scores.

(C) Length distribution of INDELs.

(D) Spectrum of nucleotide substitutions.

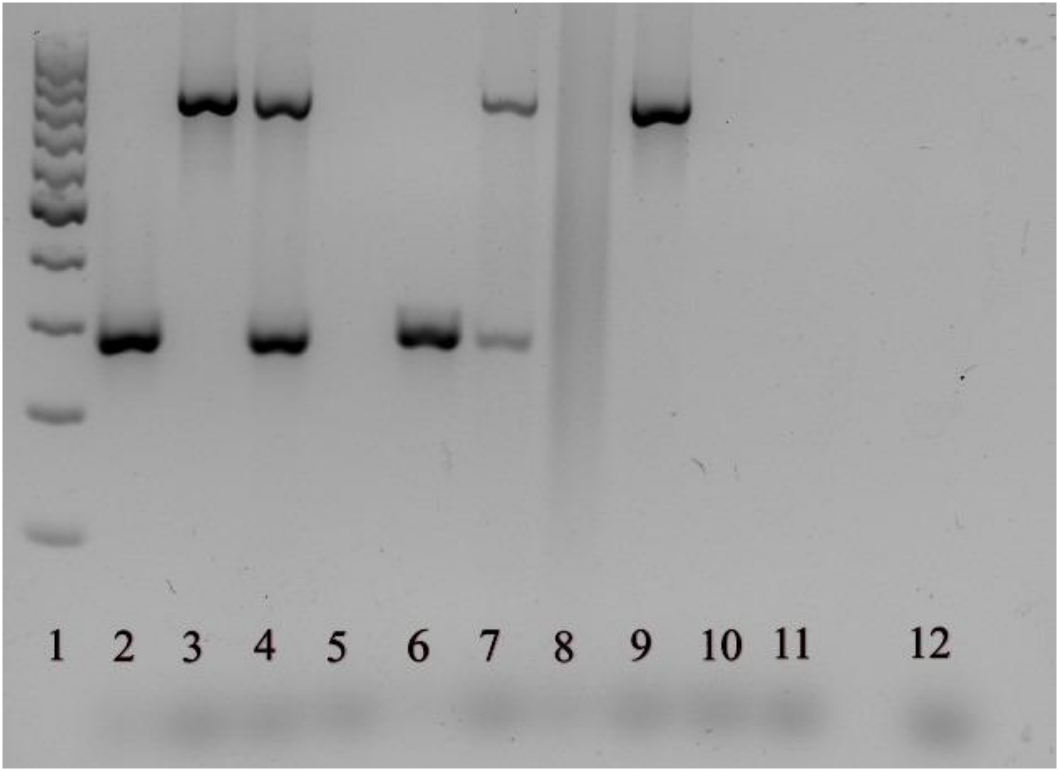

Supplementary Figure S3. Agarose gel electrophoresis (2%) showing PCR products obtained from controls and various host samples. Lane 1: molecular weight marker (100bp); 2: positive control – *Leishmania* (*Sauroleishmania*) *tarentolae* culture (strain LEM-124); 3: positive control – *Trypanosoma platydactyli* culture (isolate G9); 4: spiked DNA containing a mixture of both positive controls; 5: **Tarentolae mauritanica** negative sample; 6: *T. mauritanica* positive for *L.* (*S.*) *tarentolae*; 7: *T. mauritanica* co-infected; 8: *Podarcis siculus* negative; 9: *T. mauritanica* positive for *T. platydactyli*; 10: *Sergentomyia minuta* negative; 11: *T. mauritanica* negative; 12: no-template control (NTC).
